# Cetrotide administration in the early luteal phase in patients at high risk of ovarian hyperstimulation syndrome: A controlled clinical study

**DOI:** 10.3892/etm.2014.2005

**Published:** 2014-10-06

**Authors:** YA-QIN WANG, NAN YU, WANG-MIN XU, QIN-ZHEN XIE, WEN-JIE YAN, GENG-XIANG WU, JING YANG

**Affiliations:** 1 Reproductive Medical Center, Renmin Hospital of Wuhan University, Wuhan, Hubei 430060, P.R. China; 2 Department of Obstetrics and Gynecology, Tongji Hospital, Tongji Medical College, Huazhong University of Science and Technology, Wuhan, Hubei 430030, P.R. China

**Keywords:** gonadotropin-releasing hormone antagonist, ovarian hyperstimulation syndrome, *in vitro* fertilization, luteal phase, prevention

## Abstract

The aim of the present pilot study was to assess the feasibility and efficacy of Cetrotide administration in the early luteal phase in patients at high risk of ovarian hyperstimulation syndrome (OHSS), undergoing embryo cryopreservation following superovulation. A total of 135 patients at high risk of OHSS and undergoing embryo cryopreservation were divided into two groups. In the treatment group (n=39), the patients received daily subcutaneous injections of 0.25 mg Cetrotide between days 1 and 5 following ooctye retrieval, and volume expansion and symptomatic treatment were also provided. In the control group (n=96), the patients received routine treatments, including volume expansion therapy. The serum steroid hormone concentrations of the patients were measured on days 2, 5 and 8 following ooctye retrieval, while the incidence of moderate or severe OHSS, self-evaluated clinical symptoms and various clinical indicators were recorded. The serum estradiol (E_2_), luteinizing hormone and progesterone levels in the treatment group on days 2, 5 and 8 following oocyte retrieval were not found to differ significantly when compared with the patients in the control group (P>0.05). The incidence of severe OHSS did not differ significantly between the two groups (P>0.05). The average length of hospital stay and length of luteal phase were not found to be significantly different between the treatment and control groups (P>0.05). In conclusion, Cetrotide injections in the early luteal phase did not alter the serum steroid levels of patients at high risk of OHSS undergoing embryo cryopreservation, and were unable to reduce the incidence of severe early OHSS. However, further randomized studies are required to evaluate the effectiveness of Cetrotide in the prevention of OHSS.

## Introduction

Ovarian hyperstimulation syndrome (OHSS) is a serious iatrogenic complication that may occur following ovarian stimulation/superovulation. Symptoms include hemoconcentration, pleural effusion, hypercoagulation and multiple organ dysfunction, while severe cases of OHSS can be life-threatening (1,2). An increasing incidence of OHSS has been observed due to the rapid development of assisted reproductive technologies and the widespread application of ovulation-induction drugs (3). Despite numerous years of clinical experience, the pathophysiology of OHSS remains obscure. Delaying embryo transfer with embryo cryopreservation reduces the occurrence of pregnancy-associated late OHSS; however, there are still no precise methods to completely eliminate the incidence of human chorionic gonadotrophin (HCG)-induced severe early-onset OHSS.

Gonadotropin-releasing hormone antagonist (GnRH-ant) has been widely used in the past two decades in *in vitro* fertilization-embryo transfer (IVF-ET) to prevent luteinizing hormone (LH) surge and the suppression of estradiol (E_2_) levels. The use of GnRH-ant has been associated with a significantly lower incidence of OHSS and E_2_ concentrations as compared with GnRH agonist (GnRH-a) (4). It has previously been reported that luteal-phase GnRH-ant administration prevents patient hospitalization for patients with established severe early-onset OHSS and results in the quick regression of the syndrome on an outpatient basis (5,6). However, the LH values fall rapidly in the luteal phase of the stimulated cycles, and it remains to be determined whether the exogenous suppression of LH levels in the luteal phase is necessary. Furthermore, whether luteal-phase GnRH-ant administration can block the pathogenesis of OHSS and reduce the risk of severe OHSS has yet to be verified.

In the present study, Cetrotide, a GnRH-ant, was administered to patients at high risk of OHSS, in whom embryo transfer was canceled. The efficacy of Cetrotide in the prevention and treatment of early-onset OHSS in patients undergoing embryo cryopreservation was subsequently examined.

## Materials and methods

### Patients

A perspective, nonrandom, case-controlled study was performed at the Reproductive Medical Center, Renmin Hospital of Wuhan University (Wuhan, China) between January 2012 and June 2013. A total of 135 patients receiving IVF-ET treatment were included in the study. All participating patients met the following criteria: (i) Number of retrieved oocytes was ≥25; (ii) mean number of follicles with a diameter of >14 mm was ≥25; (iii) serum E_2_ concentrations of ≥8,000 pg/ml; (iv) ovarian diameter on the day of ovum retrieval of >10 cm; and (v) presentation of evident symptoms of OHSS on the day of aspiration. Counseling was provided to all the individuals recruited regarding the high risks and symptoms of OHSS, and all the patients agreed to cancel the fresh embryo transfer. The cases were permitted to enter the study only once. The study protocol was approved by the Ethical Research Committee of Renmin Hospital of Wuhan University, and patients were included in the study following the provision of written consent.

### Stimulation protocol and IVF procedure

In all the cases, a long mid-luteal GnRH-a protocol was adopted for superovulation. Downregulation was performed with daily subcutaneous administration of the GnRH-a, triptorelin (0.1 mg; Ferring Pharmaceuticals, Kiel, Germany), beginning on day 21 of the previous menstrual cycle, as confirmed by a blood test. After 2–3 weeks of downregulation, confirmed by a blood test and ultrasound, gonadotropin (Gn; Gonal-F; 75 IU; Merck Serono, Darmstadt, Germany) was administered intramuscularly at 150–225 IU/day, beginning on days 5–8 of the menstrual cycle. The Gn dose was adjusted according to the ovarian response. All the patients were monitored using transvaginal ultrasound and the serum E_2_ concentrations during superovulation. Final oocyte maturation was achieved by the administration of 6,000–8,000 IU HCG (1,000 IU; Lizhu Pharmaceuticals, Zhuhai, China), as soon as three or more follicles of ≥17 mm were observed by ultrasound. Transvaginal oocyte aspiration was performed 36 h later by an ultrasound-guided follicle puncture.

### Grouping and intervention

All the patients received routine preventive intravenous volume expansion therapy on the day of oocyte retrieval, and all embryos were cryopreserved due to the high risk of OHSS and/or presence of severe early OHSS. The patients were divided into two groups after being informed of the two treatment options. The treatment group (n=39) received subcutaneous injections of Cetrotide (0.25 mg/day; Merck Serono) for five consecutive days, beginning on the day following oocyte retrieval, while the control group (n=96) received no additional medication.

Blood samples were collected from all the patients on days 2, 5 and 8 following oocyte retrieval, and the serum E_2_, LH and progesterone (P4) levels were measured. The general patient information, medication prescribed for any untoward signs associated with IVF, embryonic condition, hematocrit (HCT), albumin (Alb) levels, pleural effusion, urine output and other patient parameters were monitored and recorded. In addition, the length of hospital stay, the performance of paracentesis and the amount of Alb transfused were recorded. The patients were followed-up until menstruation, for a maximum of 16 days.

The diagnostic criteria of OHSS were determined according to the classification of Golan *et al* (7). Patients with mild OHSS presented with symptoms of mild abdominal distension and discomfort, possibly accompanied by nausea, vomiting and diarrhea, and an ovarian diameter of ≤5 cm. Moderate OHSS was defined as an aggravation of the aforementioned symptoms, associated with a weight gain of >4.5 kg, ascites identified by ultrasound examination and an ovarian diameter of 5–10 cm. Severe OHSS was defined as marked ascites and/or hydrothorax, HCT >45%, white blood cell count >15,000/mm^3^, dyspnea, oliguria or abnormal liver function tests and large ovaries (>10 cm maximum diameter).

### Statistical analysis

Statistical analysis was performed using SPSS 17.0 statistical software (SPSS, Inc., Chicago, IL, USA), according to the intention-to-treat principle. All the analyses were two-sided and tested at the 5% significance level; P<0.05 was considered to indicate a statistically significant difference. Continuous variables were analyzed using the F-test in the case of normal distribution. The results of the two groups were compared using the t-test or Mann-Whitney U-test for parametric and nonparametric data, respectively. Qualitative variables were compared using the χ^2^ test with Yates correction or Fisher’s exact test. In the present study, serum P4 levels of >60 ng/ml were considered to be 60 ng/ml exactly, due to a lack of further testing to establish the exact values.

## Results

### Comparison of general information between the two groups

Age, body mass index (BMI), number of cases of polycystic ovary syndrome (PCOS), duration of infertility, baseline follicle-stimulating hormone levels, baseline E_2_ levels, duration of Gn stimulation and Gn dose were compared between the treatment and control groups. However, no statistically significant differences were observed for any of the parameters (P>0.05; Table I). In addition, no statistically significant differences were identified between the two groups with regard to the mean E_2_ concentration on the day of HCG administration, the number of follicles with a diameter of ≥14 mm, the number of oocytes retrieved, and the fertilization, cleavage and good embryo rates (P>0.05; Table II).

### Comparison of serum steroid hormone levels on different days after oocyte retrieval between the two groups

**Figure 1:**
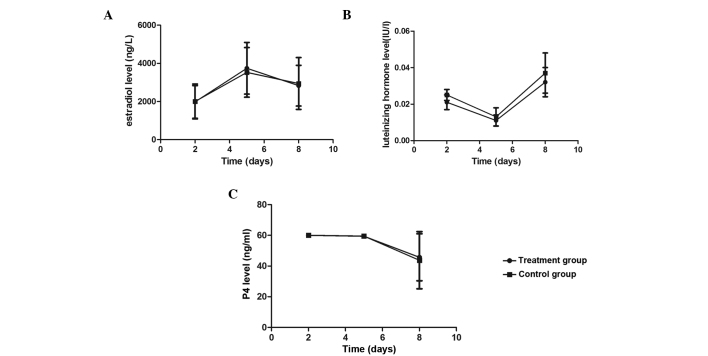
Comparison of serum steroid hormone levels on days 2, 5 and 8 following oocyte retrieval between the treatment and control groups. Concentration of serum (A) estradiol, (B) luteinizing hormone and (C) progesterone (P4).

**Figure 2:**
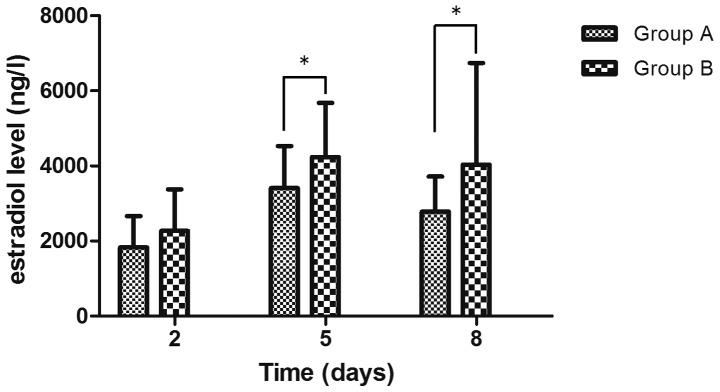
Comparison of the estradiol levels between patients with mild OHSS (Group A) and moderate or severe OHSS (Group B) in the control group at days 2, 5 and 8 following oocyte retrieval. ^*^P<0.05, vs. Group B. OHSS, ovarian hyperstimulation syndrome.

The serum E_2_, LH and P4 levels were measured on days 2, 5 and 8 following oocyte retrieval. The results revealed that the serum E_2_ levels of the two patient groups increased on days 2 and 5, while an evident decrease was observed on day 8. By contrast, the serum LH levels remained at a relatively low level on days 2,5 and 8 (<0.1 IU/l). The serum P4 levels were found to be >60 ng/ml on days 2 and 5, but a decrease was observed on day 8. No statistically significant differences were observed in the serum E_2_, LH and P4 levels between the treatment and control groups (P>0.05; Fig. 1) When comparing the serum E_2_ levels of the control patients with mild OHSS and moderate/severe OHSS on days 2, 5 and 8, the moderate or severe OHSS patients were shown to have significantly higher serum E_2_ levels (P<0.05) and a slower decline in the serum E_2_ level (Fig. 2).

### Comparison of OHSS outcome between the two groups

All the patients were followed-up until their next menses. Among the 39 patients in the treatment group, 19 cases of mild OHSS, 13 cases of moderate OHSS and seven cases of severe OHSS were identified. In the seven patients with sever OHSS, paracentesis was performed for the drainage of abdominal fluid. In the treatment group, the mean length of hospital stay was 7.0±2.8 days and the mean interval between oocyte retrieval and the onset of the next menses was 10.7±2.4 days. Among the 96 patients of the control group, 50 cases of mild OHSS, 25 cases of moderate OHSS and 21 cases of severe OHSS were identified, with 19 patients undergoing paracentesis. In the control group, the mean length of hospital stay was 7.3±3.5 days and the mean interval between oocyte retrieval and the next menses was 11.3±3.0 days. No statistically significant difference was observed in the incidence of severe OHSS between the treatment (18%) and control groups (21.8%; P>0.05). The mean length of hospital stay for the treatment group was slightly shorter compared with the control group; however, the difference was not statistically significant. Furthermore, no statistically significant difference was observed between the two groups in the number of days between oocyte retrieval and the onset of the next menses (P>0.05; Table III). Notably, no serious adverse complications were observed in either group.

## Discussion

Superovulation is a significant component of the IVF-ET process, aiming to provide numerous healthy ova and embryos. However, superovulation also increases the risk of OHSS. The main pathological features of OHSS include an increased ovarian volume and increased systemic capillary proliferation and permeability, resulting in fluid exudation, hemoconcentration and electrolyte imbalance (8,9). Although the pathogenesis of OHSS is not entirely clear, OHSS is known to be self-limiting, transient and occasionally lethal. Effectively preventing and reducing the occurrence of OHSS has become an important topic in assisted reproductive technology. The primary preventive measures include the identification of populations at high risk of OHSS and the performance of ovarian stimulation with caution. A secondary preventive measure is immediate action for early control or mitigation of OHSS development in the event of an over-reaction or tendency towards OHSS (10,11). For patients at high risk of OHSS, freezing all the embryos can effectively avoid the occurrence of late-onset OHSS. However, no effective countermeasure exists for moderate and severe early-onset OHSS, with the exception of conventional volume expansion and other symptomatic treatments. In previous studies, supplementation with cabergoline, letrozole, calcium or other drugs has been reported following ovum retrieval for the prevention and treatment of early-onset OHSS (12–16).

Cetrotide is a type of GnRH-ant that has been widely used in recent years for ovulation induction in IVF. Cetrotide rapidly binds to GnRH receptors in the anterior pituitary, inhibiting Gn release and quickly suppressing the endogenous LH surge during controlled ovarian hyperstimulation. Previous studies have demonstrated that, compared with GnRH-a protocols, GnRH-ant protocols significantly lower the serum E_2_ levels and the incidence of OHSS on the day of HCG administration (17). Hill *et al* (18) demonstrated that, even with a long-term GnRH-a protocol, the daily injection of 0.25 mg GnRH-ant prior to HCG stimulation could effectively suppress the increase in serum E_2_ levels when the serum E_2_ concentration was >4,000 pg/ml. In 2007, Lainas *et al* (5) first reported the use of an antagonist protocol for the treatment of three patients with PCOS accompanied by early-onset OHSS, with cancellation of fresh embryo transfer. Ganirelix was administered on day 3 following ovum retrieval at 0.25 mg/day for seven consecutive days. Outpatient follow-ups revealed that following the treatment, the HCT, white blood cell count, ovarian volume and ascites of the patients were significantly reduced. In addition, small sample studies performed by Hosseini *et al* (19) and Bonilla-Musoles *et al* (6) revealed that the symptoms of OHSS and the degree of severity significantly decreased following the administration of a GnRH-ant to severe early-onset OHSS patients during the luteal phase. Thus, GnRH-ant administration during the luteal phase was hypothesized to be potentially applicable for the prevention and treatment of early-onset OHSS (6,19).

Based on the aforementioned studies, the present study examined the efficacy of GnRH-ant administration during the early luteal phase in patients at high risk of early-onset OHSS who had undergone embryo cryopreservation. All the patients were at high risk of OHSS due to the relatively large number of ova retrieved during each IVF cycle, and exhibited a relatively high E_2_ level. No statistically significant differences were observed between the control and treatment groups with regard to the age, BMI, total amount of Gn administered, E_2_ level on high-HCG days, number of ova retrieved, fertilization rate or number of usable embryos. Similarly, no statistically significant difference was identified in the incidence of severe OHSS between the two groups. Since the occurrence of OHSS is associated with high levels of serum steroids, the serum E_2_ levels of patients with mild OHSS on days 2, 5 and 8 following ovum retrieval were compared with patients with moderate and severe OHSS. The serum E_2_ levels of patients with moderate and severe OHSS were significantly higher and the decrease in the E_2_ levels was slower when compared with the patients with mild OHSS. Thus, the results indicate that the serum steroid level is, to a certain degree, associated with the severity of OHSS, and that embryo transfer during the IVF treatment of patients with a relatively high estrogen level on day 2 following ovum retrieval should be performed with caution. Furthermore, no statistically significant differences were observed in the E_2_, LH and P4 levels of the patients in the treatment and control groups on days 2, 5 and 8. Therefore, GnRH-ant administration during the luteal phase does not appear to affect the secretion of steroid hormones, which is consistent with the results of Asimakopoulos *et al* (20). However, coculture of granulosa lutein cells with a GnRH-a or GnRH-ant for 48 h revealed that the concentration of vascular endothelial growth factor (VEGF) in the GnRH-ant group was significantly lower compared with the GnRH-a group, and that the VEGF level was closely associated with the manifestation of OHSS (21–23).

In theory, early-onset OHSS occurs at the same time as the zenith of corpus luteum (CL) formation, and GnRH-ants are known to exert certain luteolytic effects (24). A study by Duffy *et al*, using a monkey model, revealed a decrease in P4 levels following antide administration during the luteal phase, and the next menstrual cycle began earlier than usual (25). The CL was removed 10 days following the antide treatment and was found to weigh less than the respective control. In addition, a reduction in the number of luteal cells was observed. By contrast, Ortmann *et al* demonstrated that a low-dose of GnRH-ant was unable to exert an antagonistic effect locally in the ovary, despite GnRH receptor expression being demonstrated in the ovary (26). In the present study, the serum LH and P4 levels of the two groups were not found to differ significantly. In addition, follow-up examination did not reveal a statistically significant difference in the interval between ovum retrieval and the onset of the next menses, which is the duration of the luteal phase. When compared with the control group, the mean length of hospital stay and the incidence of paracentesis for the moderate and severe OHSS patients in the treatment group were not found to be significantly reduced. Therefore, OHSS is hypothesized to induce a cascade of effects following activation by HCG, and the application of a single GnRH-ant at a late stage does not effectively treat severe OHSS. Preventing OHSS is preferable, and the best preventive measure appears to be mild stimulation, which reduces the incidence of adverse reactions.

In conclusion, the current study presents the preliminary results of a prospective, nonrandomized clinical study. Cetrotide administration of 0.25 mg/day for five consecutive days during the early luteal phase was not found to significantly improve the clinical conditions of patients at high risk of severe early-onset OHSS. However, the number of patients in the present study was limited, and no experiments were performed to compare the effects at different dosing, timing and duration of treatment, or different types of GnRH-ant. Therefore, elucidation of the efficacy of GnRH-ant administration during the luteal phase for the prevention and treatment of early-onset moderate and severe OHSS merits further large-scale, multicenter random-controlled trials.

## Figures and Tables

**Table I:** Comparison of general information between the control and treatment groups.

Parameter	Treatment group (n=39)	Control group (n=96)	P-value
Age, years	29.9±4.2	30.1±4.0	>0.05
BMI, kg/m^2^	21.7±3.0	21.4±2.9	>0.05
PCOS cases, n	5	12	
Duration of infertility, years	4.6±3.2	4.3±3.5	>0.05
Baseline FSH, IU/l	5.9±1.8	6.1±1.6	>0.05
Baseline E_2_, pg/ml	50.9±17.8	48.2±18.2	>0.05
Duration of Gn administration, days	10.8±1.5	11.0±1.6	>0.05
Gn dose, IU	25.3±6.5	25.7±6.4	>0.05

Values are presented as the mean ± standard deviation, unless otherwise stated. P<0.05 was considered to indicate a statistically significant difference. BMI, body mass index; PCOS, polycystic ovary syndrome; FSH, follicle-stimulating hormone; E_2_, estradiol; Gn, gonadotropin.

**Table II:** Effect of treatment on *in vitro* fertilization parameters.

Fertilization parameter	Treatment group (n=39)	Control group (n=96)	P-value
Estradiol, pg/ml^a^	9349.3±2391.8	8837.1±2885.9	>0.05
Follicles with a diameter of >14 mm, n	30.6±7.3	31.1±6.9	>0.05
Ova retrieved, n	27.1±6.2	26.4±6.1	>0.05
Fertilization rate, %	81.1	83.2	>0.05
Cleavage rate, %	98.3	97.9	>0.05
Good embryo rate, %	63.1	61.9	>0.05

a Serum estradiol concentration on the day of human chorionic gonadotropin administration.

Values are presented as the mean ± standard deviation, unless otherwise stated. P<0.05 was considered to indicate a statistically significant difference.

**Table III:** Comparison of OHSS outcome between the control and treatment groups.

Parameter	Treatment group (n=39)	Control group (n=96)	P-value
Paracentesis, n (%)	7 (17.9)	19 (19.8)	>0.05
Length of hospital stay, days	7.0±2.8	7.3±3.5	>0.05
Severity of OHSS, n (%)			
Mild	19 (48.7)	50 (52.1)	>0.05
Moderate	13 (33.3)	25 (26.1)	>0.05
Severe	7 (18.0)	21 (21.8)	>0.05
Complications, n (%)	0 (0)	0 (0)	
Luteal phase, days^a^	10.7±2.4	11.3±3.0	>0.05

a Luteal phase refers to the interval between oocyte retrieval and the next menstrual cycle.

Values are presented as the mean ± standard deviation, unless otherwise stated. P<0.05 was considered to indicate a statistically significant difference. OHSS, ovarian hyperstimulation syndrome.
